# Role of Local Treatment to the Prostate in Patients With de Novo Low-volume Metastatic Hormone-sensitive Prostate Cancer Receiving Androgen Receptor Pathway Inhibitors

**DOI:** 10.1016/j.euros.2026.03.010

**Published:** 2026-03-30

**Authors:** Philipp Mandel, Mike Wenzel, Felix Chun, Markus Graefen, María Natalia Gandur Quiroga, Alejo Rodriguez-Vida, Maria T. Bourlon, Zin W. Myint, Laura Bernal, Yüksel Ürün, Ray Manneh Kopp, Maria José Juan Fita, Martin Ignacio Zapata Laguado, Thomas Büttner, Ondrej Fiala, Kazutoshi Fujita, Haoran Li, Jindrich Kopecky, Marc R. Matrana, Martin Bögemann, Francesco Carrozza, Anca Zgura, Tomas Buchler, Diana Matthews, Sati Coskun Yazgan, Edoardo Lenci, Jawaher Ansari, Fernando Sabino Marques Monteiro, Andrey Soares, Alessandro Rizzo, Francesco Ciccimarra, Francesco Massari, Abdul Rahman Jazieh, Matteo Santoni

**Affiliations:** a Department of Urology, Goethe University Frankfurt, University Hospital, Frankfurt am Main, Germany; b Martini-Klinik Prostate Cancer Center & Department of Urology, University Hospital Hamburg-Eppendorf, Hamburg, Germany; c Instituto de Oncología Ángel H. Roffo, Buenos Aires, Argentina; d Hospital del Mar, Barcelona, Spain; e Instituto Nacional de Ciencias Médicas y Nutrición Salvador Zubirán, Mexico City, Mexico; f Division of Medical Oncology, Markey Cancer Center, University of Kentucky, Lexington, KY, USA; g Clínica Universitaria Colombia Sanitas - Clínica Marly, Colombia; h Medical Oncology, Ankara University Medical Faculty, Ankara, Turkey; i Clinical Oncology, Sociedad de Oncología y Hematología del Cesar, Valledupar, Colombia; j Medical Oncology, Fundación Instituto Valenciano de Oncología, Valencia, Spain; k Internal Medicine, Universidad El Bosque, Bogotá, Colombia; l Department of Urology, University Hospital Bonn (UKB), Bonn, Germany; m Department of Oncology and Radiotherapeutics, Faculty of Medicine, University Hospital in Pilsen, Charles University, Pilsen, Czech Republic; n Biomedical Center, Faculty of Medicine in Pilsen, Charles University, Pilsen, Czech Republic; o Department of Urology, Kindai University, Faculty of Medicine, Kindai, Japan; p Division of Medical Oncology, Department of Internal Medicine, University of Kansas Cancer Center, Westwood, KS, USA; q Department of Clinical Oncology and Radiotherapy, University Hospital Hradec Kralove, Hradec Kralove, Czechia; r Department of Internal Medicine, Hematology/Oncology, Ochsner Medical Center, New Orleans, LA, USA; s Department of Urology, University Hospital Münster, Münster, Germany; West German Cancer Center, Münster, Germany; t Department of Oncology-Hematology, Ospedale Santa Maria delle Croci, UOC Oncologia Ravenna, AUSL della Romagna, Italy; u Department of Oncology-Radiotherapy, Prof. Dr. Alexandru Trestioreanu Institute of Oncology, “Carol Davila,” University of Medicine and Pharmacy, Bucharest, Romania; v Department of Oncology, Second Faculty of Medicine, Charles University and University Hospital Motol, Prague, Czech Republic; w Velindre Cancer Centre, Velindre Road, Whitchurch, Cardiff, Wales, UK; x Medical Oncology Unit, Azienda Ospedaliera Ospedali Riuniti Marche Nord, Pesaro, Italy; y Medical Oncology, Tawam Hospital, Al Ain, United Arab Emirates; z Latin American Cooperative Oncology Group - LACOG, Porto Alegre, Brazil; aa Hospital Sírio-Libanês, Brasília, DF, Brazil; bb Hospital Israelita Albert Einstein, São Paulo, SP, Brazil; cc S.S.D. C.O.r.O. Bed Management Presa in Carico, TDM, IRCCS Istituto Tumori “Giovanni Paolo II”, Bari, Italy; dd Medical Oncology, IRCCS Azienda Ospedaliero-Universitaria di Bologna, Bologna, Italy; ee Department of Medical and Surgical Sciences (DIMEC), University of Bologna, Bologna, Italy; ff Cincinnati Cancer Advisors, Cincinnati, OH, USA; gg Medical Oncology Unit, Macerata Hospital, Macerata, Italy

**Keywords:** Prostate cancer, mHSPC, Low-volume, ARPI, Local therapy

## Abstract

**Background and objective:**

Local treatment (LT) to the prostate demonstrated better cancer-control outcomes in combination with androgen deprivation therapy (ADT) monotherapy for patients with low-volume metastatic hormone sensitive prostate cancer (mHSPC). However, the association of LT with outcomes in patients receiving ADT plus androgen receptor pathway inhibitors (ARPI) across different mHSPC subtypes is under debate.

**Methods:**

Relying on the multicentric international ARON-3 database, patients with de novo low-volume mHSPC undergoing ARPI treatment were selected. Stratification was made according to LT vs. no LT with the primary endpoint of time on treatment (ToT) and overall survival (OS).

**Key findings and limitations:**

Of 454 patients with de novo low-volume mHSPC, LT was administered in addition to ARPI in 18%. In the 6-mo landmark cohort, ToT was longer in patients who received additional LT, although the association did not reach statistical significance (hazard ratio [HR]: 0.54, 95% confidence interval [CI]: 0.27–1.10, *p* = 0.088). The restricted mean survival time (RMST) at 36 mo reported a difference of 2.76 mo (95% CI: 0.32–6.58, *p* = 0.031) in the LT group compared with the no-LT group. OS was significantly longer in patients receiving ARPI and LT compared with ARPI alone (HR: 0.09, 95% CI: 0.01–0.64, *p* = 0.016). The RMST at 36 mo reported a difference of 4.67 mo (95% CI: 3.18-6.17, *p* < 0.001) in favor of the LT group. In the ridge regression, LT remained the only statistically significant predictor of ToT and OS.

**Conclusions and clinical implications:**

The current study suggests that adding LT to ARPIs in patients with de novo low-volume mHSPC may be associated with improved ToT and OS. The addition of LT to ARPI as the backbone of therapy may be considered in patients presenting with de novo low-volume mHSPC.

## Take Home Message

The current study provides robust real-world evidence that adding local treatments (LT) to androgen deprivation therapies (ARPIs) in patients with de novo low-volume metastatic hormone-sensitive prostate cancer (mHSPC) may be associated with improved time on treatment and overall survival. Sensitivity analyses across specific subgroups of patients with Eastern Cooperative Oncology Group Performance Status 0-1 and those aged <70 yr were consistent with these findings. LT, in addition to ARPI, as the backbone of therapy, might be discussed with eligible patients presenting with de novo low-volume mHSPC disease as part of a multimodal treatment approach.

ADVANCING PRACTICE **What does this study add?**

The current study adds important knowledge to the limited data on local therapy in addition to intensified antihormonal therapy with androgen receptor pathway inhibitors (ARPIs) in patients with de novo low-volume metastatic prostate cancer. Local therapy may be associated with an oncological benefit in a large multinational dataset. Therefore, the addition of local treatment to the “backbone” of systemic therapy with ARPIs might be discussed with eligible patients.

**Clinical Relevance**

Randomized trials suggest that adding local therapy to androgen deprivation therapy may provide a clinical benefit in men with metastatic prostate cancer, particularly in those with a lower metastatic burden. However, with the advent of more effective upfront systemic combination therapies, the magnitude of this benefit may be reduced. This study offers additional insight into the role of such combination approaches. Importantly, any potential advantage of local therapy must be carefully weighed against the risk of added toxicity, as well as the associated costs and resource utilization. Associate Editor: Roderick C.N. van den Bergh.

**Patient Summary**

In this report, we examined the impact of a local treatment in addition to intensified antihormonal therapy in patients with a low metastatic burden at the time of diagnosis. Local therapy may be associated with prolonged survival. We conclude that local therapy might be an option for eligible patients.

## 1 Introduction

For metastatic hormone-sensitive prostate cancer (mHSPC), several life-prolonging systemic treatment options are currently available. These consist of androgen receptor pathway inhibitors (ARPI), taxane-based chemotherapy, or their combination in addition to conventional androgen deprivation therapy (ADT) [2], [3], [4], [5], [6], [7]. Currently, the combination treatment with ARPI, such as apalutamide, enzalutamide (or darolutamide), or abiraterone plus ADT, has become the standard of care in patients with Chemohormonal Therapy versus Androgen Ablation Randomized Trial for Extensive Disease in Prostate Cancer (CHAARTED) low-volume mHSPC, based on data from several prospective randomized phase III trials, whereas a further intensification with triplet-therapy can be administered in patients with high-volume disease [3], [4], [5], [6], [7], [8], [9], [10], [11], [12], [13], [14], [15].

Despite systemic treatment in mHSPC, previous prospective data, especially from the STAMPEDE trial and the HORRAD trial for radiotherapy (RT), and from two smaller trials for radical prostatectomy (RP), also suggested a beneficial effect on cancer-control outcomes of local treatment (LT) to the primary prostate tumor in patients with CHAARTED low-volume mHSPC, relative to ADT alone [16], [17], [18], [19]. However, recent evidence from the randomized controlled PEACE-1 trial has demonstrated that the addition of radiotherapy to intensified systemic therapy (ADT + abiraterone) improves progression-free survival (PFS) in patients with de novo low-volume mHSPC [20].

Consequently, often two different treatment approaches consisting of local treatment (LT) to the prostate and systemic treatment with ADT plus ARPI are currently administered in clinical practice for patients with de novo low-volume mHSPC, as both showed favorable cancer-control outcomes compared to ADT monotherapy. However, evidence on the association between LT and intensified systemic treatment for patients with de novo low-volume mHSPC is still under debate and has never been evaluated in a large real-world cohort so far. Herein, relying on the multicentric, international ARON-3 collaboration real-world cohort, we evaluated the association between LT to the prostate and outcomes in patients with de novo low-volume mHSPC undergoing systemic ARPI treatment, relative to patients without further LT. Specifically, we hypothesized that important cancer-control outcome differences may exist between these specific patient cohorts.

## 2 Patients and methods

### 2.1 Study design and population

This study focused on adult patients who received ADT plus ARPI between January 1, 2019, and November 30, 2024, for de novo low-volume mHSPC, defined as in the CHAARTED trial [2]. Given the retrospective, observational, nonrandomized design, results are presented as associations, and residual confounding cannot be excluded.

Participants were selected from the ARON-3 database. Clinical and pathological data were gathered from medical and pathological reports across 28 oncology centers in 13 different countries (Fig. S1). Information collected included age, tumor histology, Eastern Cooperative Oncology Group Performance Status (ECOG-PS), number and location of metastatic sites, RP or RT, ARPI dosage and duration, and prostate-specific antigen (PSA) response during treatment. Patients with incomplete clinical or outcome data were excluded from the analysis.

### 2.2 Study objectives

The primary aim of this study was to assess real-world outcomes associated with LT on the primary tumor in patients with de novo low-volume mHSPC who received ADT plus ARPI. Metastases were detected by conventional imaging or molecular imaging. Specifically, the two coprimary endpoints were the time on treatment (ToT) and overall survival (OS). ToT was defined as the period from the initiation of ADT plus ARPI until discontinuation for any reason, including treatment-related toxicity. OS was calculated from the start of ADT plus ARPI therapy to the time of death from any cause. Severe adverse events (AEs), categorized as grade ≥3 according to the Common Terminology Criteria for Adverse Events v5.0, or those leading to dose reduction or treatment interruption, were also recorded as secondary endpoints.

### 2.3 Statistical considerations

Statistical analyses and reporting followed the European Urology statistical reporting guidelines and the European Urology framework for causal inference in observational research [1], [21]. Continuous variables were summarized using the median and interquartile range, whereas categorical variables were summarized using counts and percentages. Group comparisons used c2 or Fisher's exact tests for categorical variables and the Mann–Whitney U test for continuous variables, as appropriate.

To minimize immortal-time bias, exposure to LT was defined using a 6-mo landmark. Patients who experienced the event of interest before mo 6 (death for OS; treatment discontinuation for ToT) were excluded. Patients who received LT within 6 mo of starting ADT + ARPI were classified as exposed, whereas those who did not receive LT within 6 mo were classified as unexposed; this exposure status was carried forward. Patients who had not discontinued treatment by the end of the follow-up period were censored at the date of last documented treatment administration or last clinical visit, whichever came later.

Patients who were lost to follow-up were censored on the date of their last contact. Kaplan–Meier curves were truncated when the number at risk in either group fell below five participants.

All statistical analyses were performed on the 6-mo landmark cohort.

Covariates were prespecified on causal grounds as potential confounders of the association between LT and outcomes: age, PSA at baseline, ECOG-PS, bone and lymph-node metastases, and Gleason score grade group [22]. All variables were retained in the multivariate model. According to our causal model, these variables act as common causes of both the likelihood of receiving LT and the oncological outcomes. Therefore, they represent the minimally sufficient adjustment set to control for confounding in our observational design. Restricted mean survival time (RMST) was computed at τ = 36 mo, the latest time point at which both groups had more than 10 patients at risk remaining.

RMST differences (ΔRMST) with 95% confidence intervals (CIs) were obtained.

Effect modification was explored by adding interaction terms between LT and ECOG-PS, age, and modality of LT (RT vs non–RT). Effect modification was assessed using unpenalized Cox proportional-hazards models, comparing nested models with and without each interaction using likelihood-ratio tests. The corresponding interaction *p*-values were reported.

The association between LT and outcomes was evaluated using penalized Cox proportional-hazards models (ridge penalty, α = 0) with 10-fold cross-validation for λ selection in OS and ToT. Model performance was assessed using Harrell’s concordance index (C-index) with 95% CIs, and calibration at 24 mo was evaluated using observed versus predicted survival. Age (per 10 yr) and baseline PSA (log_10_-transformed) were modeled as continuous variables using natural cubic splines (3 degrees of freedom). To facilitate interpretation, clinically meaningful contrasts were derived from the spline-based penalized Cox models by comparing specific values of age (70 vs 60 y, 80 vs 70 y) and PSA (20, 50, 100, and 200 ng/ml vs 10 ng/ml), holding other covariates at reference levels. Hazard ratios (HRs) were computed as exponentiated differences in linear predictors, and 95% CIs were obtained through nonparametric bootstrap resampling (500 iterations), refitting the penalized Cox model at each iteration.

Two-sided *p* < 0.05 values were considered statistically significant.

Given the observational design, all analyses were hypothesis-generating and should be interpreted as associations rather than causal effects.

All statistical analyses were conducted using RStudio software (v4.5.1) and the following packages: survival, glmnet, survRM2, survminer, spline.

### 2.4 Ethical considerations

Ethical approval for the ARON-3 study was granted by the Ethics Committee of the Marche Region (2024-20) and the Institutional Review Boards of all participating sites, adhering to the regulations in each respective country. The study was conducted in compliance with Good Clinical Practice guidelines and international ethical standards for biomedical research. All procedures followed the ethical principles set out in the Declaration of Helsinki for research involving human participants.

## 3 Results

### 3.1 Patient characteristics

A total of 454 patients treated with ARPI for de novo low-volume mHSPC were extracted from the ARON-3 dataset (Fig. S2). Median follow-up was 24.4 mo (Interquartile range [IQR]: 13.2.3–37.7) in the entire cohort; 31.2 mo (IQR: 13.1–39.0) for patients with LT versus 23.9 mo (IQR: 13.2.3–37.7) for patients without LT (*p* = 0.7). At the time of this analysis, 48 patients (11%) had died.

Patients and tumor characteristics are depicted in Table 1. LT to the prostate was reported in 83 patients (18%) and consisted of RT in 40 patients (9%) and RP in 43 patients (9%). In 74 patients (16%), LT was performed within 6 mo of the start of ARPI plus ADT, whereas 8 patients (2%) received LT after 6 mo from the beginning of doublet therapy. Twenty-five patients (6%) received docetaxel-based CHAARTED regimen before ARPI therapy in mHSPC. The complete list of patient characteristics is illustrated in Table 1.

**Table 1:** Clinical and pathological characteristics of patients treated with ARPI + ADT with or without local treatments. Significant *p*-values were reported in bold.

Patients	ARPI + ADT + local treatments No (%)	ARPI + ADT No (%)	*p* value
Total patients	83	371	–
Age, yr (y)			
Median [IQR]	66.5 [60–72]	72 [65–76.5]	<0.001
ECOG-PS			
0	61 (73)	245 (66)	0.175
1	18 (22)	117 (32)	
≥2	4 (5)	9 (2)	
Grade Group at initial diagnosis			
1–3	21	111	0.527
4–5	62 (75)	260 (70)	
Metastatic sites			
Distant Lymph nodes only	55 (66)	232 (63)	0.768
Bone metastasis ± lymph nodes	47 (57)	299 (81)	<0.001
Radiation therapy on the primary tumor	40 (48)	–	–
Prostatectomy	43 (52)	–	–
First-line treatment for mHSPC			
Apalutamide + ADT	45 (54)	129 (35)	0.024
Enzalutamide + ADT	15 (18)	87 (23)	
Abiraterone + ADT	23 (28)	155 (42)	
Docetaxel therapy	4 (5)	21 (6)	1.000
PSA value before starting ADT + ARPI			
Median (ng/ml) [IQR]	16.8 [5.46–44.83]	66 [11.69–431.25]	<0.001

ADT = androgen deprivation therapy; ARPI = androgen receptor pathway inhibitor; ECOG-PS = Eastern Cooperative Oncology Group performance status; IQR = interquartile range; mHSPC = metastatic hormone-sensitive prostate cancer.

### 3.2 Time of treatment

**Fig. 1:**
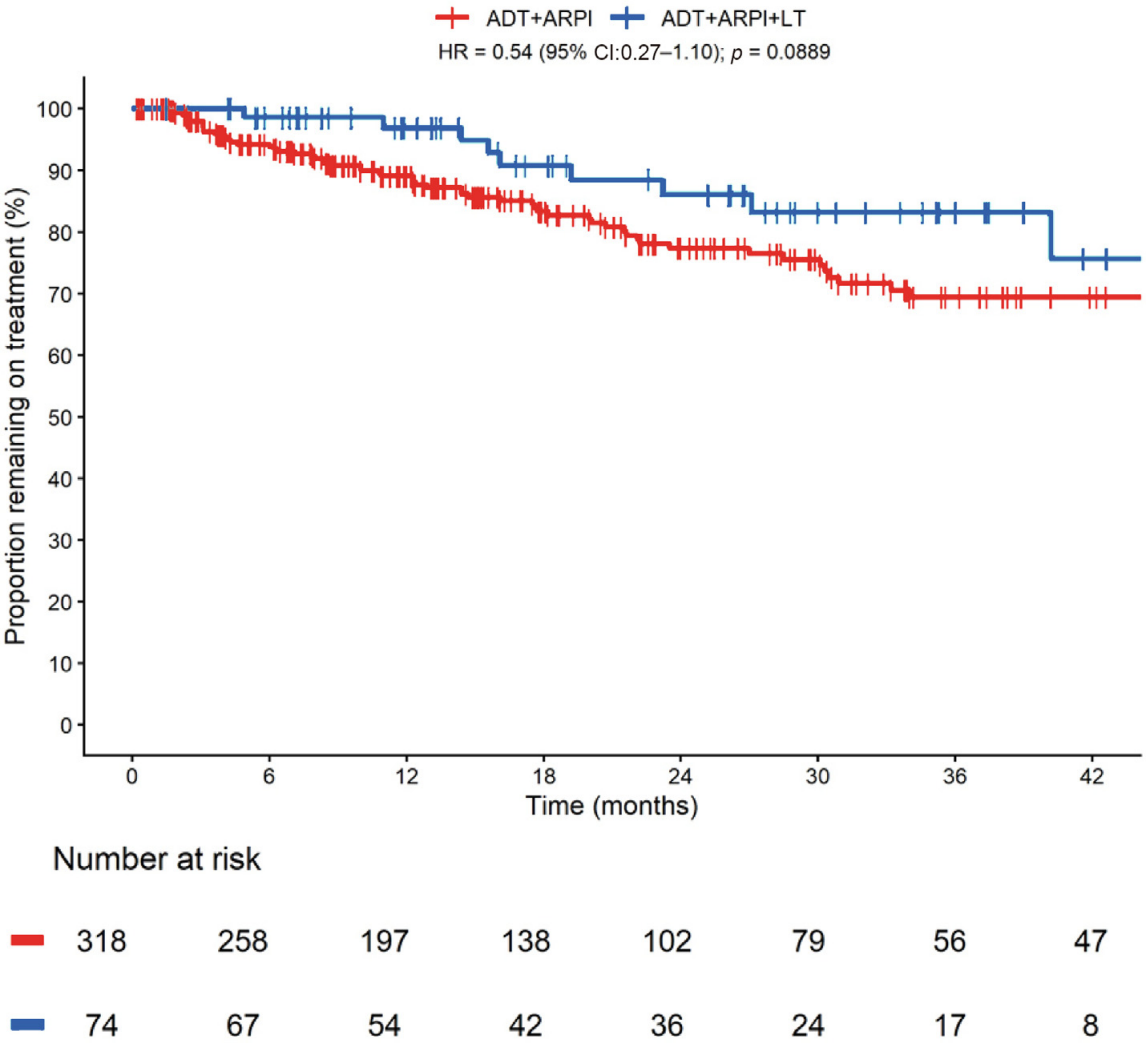
Time on Treatment in de novo low-volume mHSPC patients treated with ADT plus ARPI ± local treatments (LT, including prostatectomy and radiotherapy on primary tumor, RT). ADT = androgen deprivation therapy; ARPI = androgen receptor pathway inhibitor; CI = confidence interval; HR = hazard ratio; LT = local treatments; mHSPC = metastatic hormone-sensitive prostate cancer; RT = radiotherapy.

In the 6-mo landmark cohort, ToT was longer in patients who received additional LT, although the association did not reach statistical significance (HR: 0.54, 95% CI: 0.27–1.10, *p* = 0.088, Fig. 1). The 2y-ToT rate was 86% in the LT group versus 77% in the no-LT group. Median ToT was not reached in either group.

The RMST at 36 mo reported a difference of 2.76 mo (95% CI: 0.32–6.58, *p* = 0.031) in the LT group compared with the no-LT group.

We observed evidence of effect modification by ECOG-PS (*p* for interaction = 0.025), with a stronger association of LT with longer ToT among patients with ECOG-PS 0–1 than among those with ECOG-PS ≥2. We found no evidence of interaction with age (*p* = 0.10) or LT modality (*p* = 0.96).

In the ridge-penalized Cox regression, LT was an independent predictor of ToT (HR: 0.79, 95% CI: 0.19–0.98) (Table 2). The penalized model showed a C-index of 0.65 (95% CI: 0.55–0.68). Calibration at 24 mo was reasonable, although the model tended to underestimate absolute risk (Fig. S3)

**Table 2:** Ridge-penalized Cox regression for time on treatment

Ridge Regression	HR (95% CI)
Local treatment (yes vs no)	0.79 (0.19–0.98)
ECOG-PS (2 vs 0–1)	1.06 (0.16–5.58)
Bone metastases (yes vs no)	0.97 (0.36–1.21)
Distant lymph nodes (yes vs no)	0.92 (0.50–1.15)
Grade group 4–5 versus Grade group 1–3	1.01 (0.70–1.83)

CI = confidence interval; ECOG-PS = Eastern Cooperative Oncology Group performance status; HR = hazard ratio.

Moreover, we found no evidence that ARPI type (apalutamide, enzalutamide, abiraterone) modified the association between LT and ToT. None of the spline-based contrasts for age or PSA showed significant associations in the penalized model (Table 3).

**Table 3:** Contrasts for age and PSA in the Ridge regression model for time on treatment

	HR (95% CI)
Age (yr)	
70 vs 60	0.95 (0.59–1.18)
80 vs 70	0.95 (0.59–1.43)
PSA (ng/ml)	
20 vs 10	0.99 (0.93–1.16)
50 vs 10	0.98 (0.80–1.19)
100 vs 10	0.98 (0.66–1.20)
200 vs 10	0.99 (0.55–1.30)

CI = confidence interval; HR = hazard ratio; PSA = prostate-specific antigen.

### 3.3 Survival analysis

**Fig. 2:**
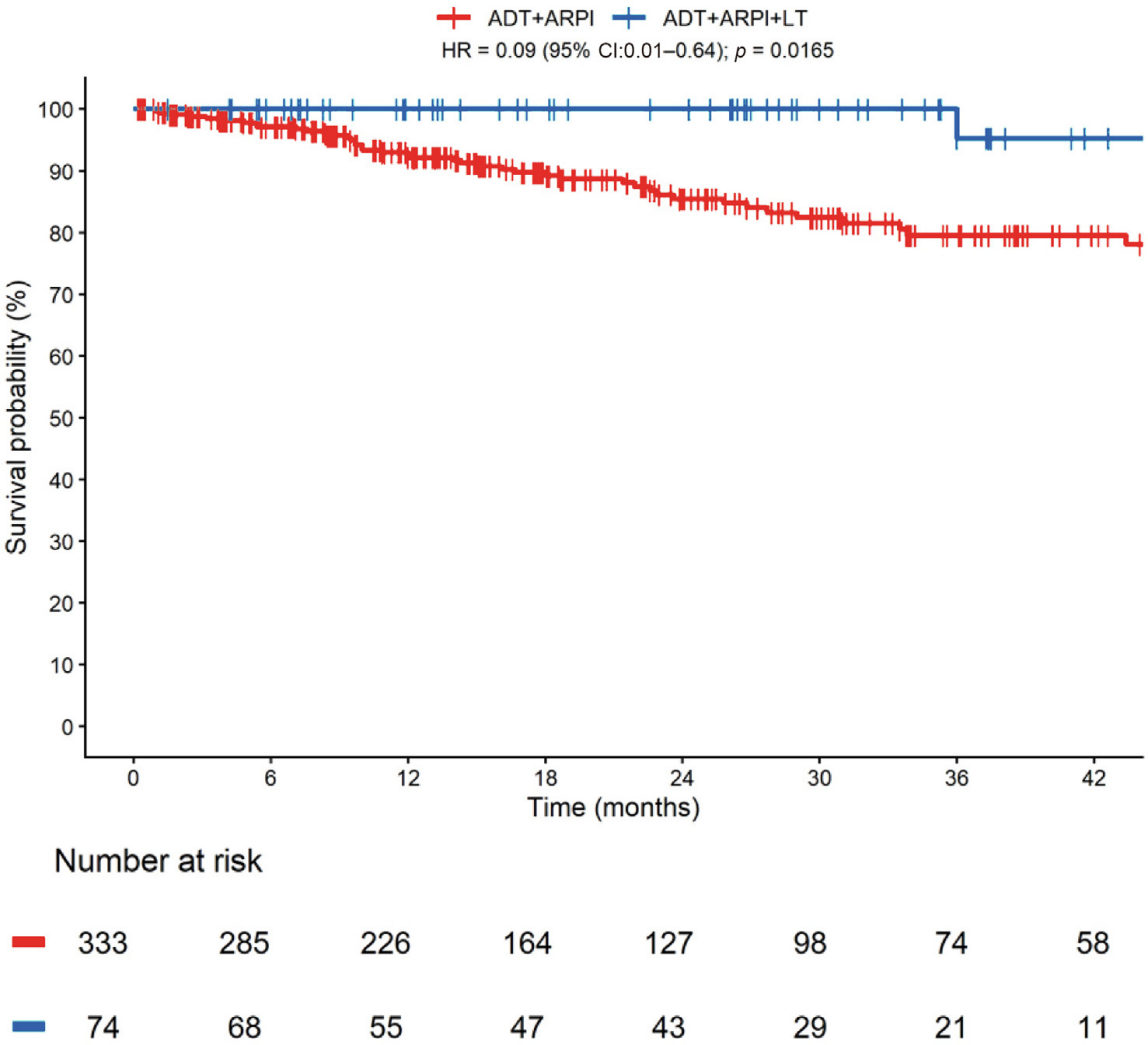
Overall Survival in de novo low-volume mHSPC patients treated with ADT plus ARPI ± local treatments (LT). ADT = androgen deprivation therapy; ARPI = androgen receptor pathway inhibitor; CI = confidence interval; HR = hazard ratio; LT = local treatments; mHSPC = metastatic hormone-sensitive prostate cancer.

In the 6-mo landmark cohort, OS was significantly longer in patients receiving ARPI and LT compared with ARPI alone (HR: 0.09, 95% CI: 0.01–0.64, *p* = 0.016, Fig. 2), with a corresponding 2y-OS rate of 100% vs 85%.

The RMST at 36 mo reported a difference of 4.67 mo (95% CI: 3.18–6.17, *p* < 0.001) in favor of the LT group.

We observed no evidence that OS differed by ARPI type in this cohort.

No evidence of effect modification was detected for ECOG-PS (*p* = 0.74), age (*p* = 0.09), or LT modalities (*p* = 0.21).

In the ridge regression, LT remained the only statistically significant predictor (HR: 0.48, 95% CI: 0.07–0.59) (Table 4).

**Table 4:** Ridge-penalized Cox regression for overall survival

Ridge Regression	HR (95% CI)
Local treatment (yes vs no)	0.48 (0.07–0.59)
ECOG-PS (2 vs 0–1)	1.48 (0.16–14.59)
Bone metastases (yes vs no)	1.27 (0.63–3.57)
Distant lymph nodes (yes vs no)	0.92 (0.54–1.65)
Grade group 4–5 vs Grade group 1–3	1.22 (0.79–2.95)

CI = confidence interval; ECOG-PS = Eastern Cooperative Oncology Group performance status; HR = hazard ratio.

Model performance was acceptable, with a C-index of 0.71 (95% CI: 0.63–0.71), and calibration at 24 mo was adequate (Fig. S4). None of the age or PSA contrasts were statistically significant (Table 5).

**Table 5:** Contrasts for age and PSA in the Ridge regression model for overall survival

	HR (95% CI)
Age (yr)	
70 vs 60	0.96 (0.53–1.36)
80 vs 70	1.47 (0.88–2.97)
PSA (ng/ml)	
20 vs 10	1.02 (0.98–1.33)
50 vs 10	0.98 (0.78–1.40)
100 vs 10	0.94 (0.55–1.43)
200 vs 10	0.91 (0.41–1.52)

CI = confidence interval; HR = hazard ratio; PSA = prostate-specific antigen.

### 3.4 Safety and subsequent therapies

Thirty-four patients (7%) presented severe AEs (SAEs), 4 in the LT group (4%) and 30 in patients treated with ADT plus ARPI (8%). The most frequent SAEs were fatigue (4%), rash (2%), bone fractures (1%), and hypertension (1%).

Fifty-three of the patients progressed during first-line therapy (11% of patients with LT and 12% without LT). Further treatments for metastatic castration-resistant prostate cancer (mCRPC) were: *n* = 21 docetaxel, *n* = 11 abiraterone acetate, *n* = 7 enzalutamide, *n* = 4 cabazitaxel, *n* = 4 olaparib, *n* = 6 others.

## 4 Discussion

We initially hypothesized that cancer-control outcome differences may exist in patients with low-volume de novo mHSPC receiving LT according to the STAMPEDE criteria when simultaneously receiving ARPI. We tested this hypothesis within the multinational ARON-3 database and made several important observations.

First, we observed that patient and tumor characteristics of the present study were well balanced for most baseline and tumor characteristics between the two groups, although some important differences were evident, particularly for age and the presence of bone metastases. This relative balance likely reflects the homogeneous and strict inclusion criteria of the study, which focused on de novo low-volume mHSPC. The only statistically significant difference between the two groups was the rate of bone metastases (57% vs 81%), a known prognostic factor; although we adjusted for metastatic burden in our causal model, such imbalances remain a potential source of residual confounding. This is consistent with previous studies in which the presence of bone metastases was associated with worse oncological outcomes [23]. However, patient and tumor characteristics were basically comparable to other trials that evaluate the role of systemic or LT in patients with low-volume prostate cancer [5], [16], [17], [18], [23], [24], [25]. Therefore, our results seem to be comparable to the currently available data in the literature.

Second, we made some observations regarding ToT and OS of patients with de novo low-volume mHSPC receiving ARPI ± LT. In the 6-mo landmark cohort, patients receiving LT had longer ToT than those with ARPI alone, although this association did not reach conventional statistical significance in the unadjusted Cox model, whereas RMST suggested a modest but statistically significant difference at 36 mo. The magnitude of the association between LT and ToT in our analyses was broadly comparable to the HRs for PFS or failure-free survival reported in phase II/III trials of LT in de novo low-volume disease receiving ADT alone, suggesting a similar relative effect across different systemic backbones [16], [17], [18]. One might argue that a follow-up of 24.4 mo in the present study might not be sufficient for a conclusive analysis of OS rates, also in light of the low event rate, especially in the LT group. Still, when comparing the ARPI group without LT in the present study, OS results are broadly similar to pivotal studies of the respective ARPIs [5], [22]. Therefore, these associations between LT and ARPIs may persist with longer follow-up, but confirmation in prospective studies is needed.

Third, our findings align with prior research demonstrating the oncological benefits of LT in mHSPC. The STAMPEDE and HORRAD trials previously showed that LT with RT improves PFS and failure-free survival in patients receiving ADT alone. However, the role of LT in combination with intensified systemic therapy, such as ARPI, has remained unclear. Our observational data contribute to this evidence, suggesting that LT is associated with more favorable oncological outcomes even among patients receiving ARPI, a more potent systemic therapy than ADT alone. Moreover, we compare our results directly to the results of the phase III PEACE-1 trial, where adding RT to standard of care plus abiraterone improved PFS and castration resistance-free survival, but not OS, in patients with de novo low-volume mHSPC [6], [20]. Our findings are consistent with the PEACE-1 results for ToT, but we observed an association with longer OS. Moreover, in PEACE-1, median OS for de novo low-volume mHSPC was 7.5 vs 6.9 yr for standard of care plus/minus abiraterone and with vs without RT. However, the standard of care within the PEACE-1 trial was heterogeneous and partly (50%–60%) consisted of docetaxel combined with abiraterone, which is currently not considered the standard of care and may have influenced OS outcomes. Taken together, the underlying systemic treatments differed compared to PEACE 1 and might also have influenced the results.

We performed a 6-mo landmark analysis to emulate a pragmatic target trial and minimize immortal-time bias. Within this framework, patients receiving LT within 6 mo appeared to have similar associations with ToT and OS. However, our data are insufficient to determine an optimal timing of LT or to recommend delaying local therapy in clinical practice. Our study was not powered to compare RT versus RP directly, and both modalities appeared to have broadly similar associations with oncological outcomes. Nonetheless, the findings and methods are consistent with previous studies grouping RP and RT as a cytoreductive treatment modality [18].

We also observed important findings regarding AE rates. Specifically, adding LT to ARPI was not associated with an increase in new safety signals in terms of higher SAE rates. This observation is very important, as a more intensive therapy, which shows better oncological results, might also lead to higher rates of AEs, which might lower the overall benefit and reduce quality of life. Unfortunately, our study lacks data providing information on local symptoms. A potential reduction of local complications is, besides the outlined oncologic benefit, a major argument for favoring LT in mHSPC. In the HORRAD trial, for example, a reduction of local events by almost half from 50/216 to 30/216 patients due to RT was observed (*p* = 0.04) [17]. In the LOMP trial, 2-yr local event-free survival rates were 92%, 77%, and 60% for patients with RP, RT, and no LT in mHSPC, respectively.

This study has several strengths. We used a 6-mo landmark design to minimize immortal-time bias, and we adjusted for a prespecified causal set of key baseline confounders in line with contemporary recommendations for observational research. We also applied ridge-penalized Cox models to stabilize estimates in the presence of correlated variables and improve the robustness of regression coefficients [26], [27]. Model performance was evaluated through discrimination and calibration at 24 mo, a clinically relevant time point given the median follow-up of our cohort and current guidance for survival prediction models [28].

In addition to the above-mentioned limitations, our study should be interpreted in light of the nonrandomized, retrospective design and the mid-term follow-up duration. Moreover, some missing data, as well as other unreported variables, may have influenced cancer-control outcomes, eg, the staging modality used for metastases (conventional vs molecular imaging). Patients receiving LT were younger and less likely to have bone metastases at baseline, consistent with confounding by indication. Although we attempted to mitigate this bias by adjusting for a prespecified causal set of confounders and by using penalized regression, some degree of residual confounding cannot be excluded [29], [30]. Furthermore, the surgical experience and the exact RT protocol are unknown and might differ.

ToT calibration at 24 mo suggested that the model tended to underestimate absolute risk across strata. These factors should be considered when interpreting our results.

Taken together, our real-world data suggest that adding LT to ARPIs in patients with de novo low-volume mHSPC is associated with longer ToT and OS. These findings are hypothesis-generating and support further evaluation of LT as part of multimodal treatment strategies in this population, but they should not be interpreted as definitive evidence. LT, in addition to ARPI, as the backbone of therapy, may be considered as part of multimodal treatment in decision-making for patients with de novo low-volume mHSPC, while acknowledging residual confounding and the hypothesis-generating nature of the findings. Nevertheless, further prospective studies are warranted to validate these findings and refine patient selection criteria for LT in this setting.

  ***Author contributions***: Philipp Mandel had full access to all the data in the study and takes responsibility for the integrity of the data and the accuracy of the data analysis.

  *Study concept and design*: Philipp Mandel, Matteo Santoni.

*Acquisition of data*: All authors.

*Analysis and interpretation of data*: Philipp Mandel, Mike Wenzel, Matteo Santoni.

*Drafting of the manuscript*: Philipp Mandel, Matteo Santoni.

*Critical revision of the manuscript for important intellectual content*: All authors.

*Statistical analysis*: Matteo Santoni.

*Obtaining funding*: None.

*Administrative, technical, or material support*: None.

*Supervision*: Philipp Mandel, Matteo Santoni.

*Other* (specify): None.

  ***Financial disclosures:*** Philipp Mandel reports payments or honoraria from presentations, speaker bureaus, consulting or travel expenses from Johnsen&Johnsen, Novartis, Orion, Bayer, Astellas, Ipsen, Amgen, AstraZeneca, Pfizer, MSD.

Mike Wenzel receives speaker honoraria or is consultant for Accord, Astellas, Johnsen&Johnsen, Pfizer, MSD, Astra Zeneca, Ipsen, Bayer.

Felix Chun reports advisory boards for Astellas, Bayer, Janssen, Lumenis (KOL), Molecular Health, Olympus, Pfizer and speaker honoraria from Novartis, Astellas, Astra Zeneca, Janssen, Lumenis, Olympus, Ipsen.

Markus Graefen receives speaker honoraria from or is a consultant for Intuitive Surgical, Metronic, Ipsen, Astellas, Johnson & Johnson, and Takeda.

Fernando Sabino M. Monteiro: Research support was provided by Merck Sharp Dome and Foundation Medicine. Honoraria from Janssen, Ipsen, Bristol Myers Squibb, and Merck Sharp Dome. Travel expenses by Novartis, Bayer, ADIUM, Merck and Mer Sharp Dome. Ownership: BIO, Brazilian Information Oncology. All are unrelated to this study. ORCID: 0000-0002-6621-8251.

Andrey Soares: Honoraria from Janssen, Pfizer, Bayer, Merck Serono, Novartis. Consulting or Advisory Role from Janssen, Bayer, AstraZeneca, MSD, Pfizer, Novartis. Research Funding from Bristol-Myers Squibb (Inst), Astellas (Inst), AstraZeneca (Inst). Travel, Accommodations, Expenses from Bayer, Janssen, MSD, Merck Serono. Ownership: BIO, Brazilian Information Oncology. All are unrelated to this study. Nonfinancial with no other potential conflicts of interest were reported. ORCID: 0000-0003-4980-6729.

Diana Matthews received honoraria from Janssen, Astellas and Bayer for consultations and lectures unrelated to this project.

Ondřej Fiala received honoraria from Novartis, Janssen, Merck, BMS, MSD, Pierre Fabre, and Pfizer for consultations and lectures unrelated to this project.

Tomas Buchler has received payment or honoraria for lectures, presentations, speakers’ bureaus, manuscript writing, or educational events from Bristol Myers Squibb, Ipsen, Roche, Astellas, Merck, Eisai, Merck Sharp Dohme, Novartis, AstraZeneca, Janssen, and Pfizer, all unrelated to this project.

Kazutoshi Fujita received honoraria from Jannsen and Astellas.

Martin Boegemann reports serving in an advisory role for Pfizer und receiving honoraria by, Bristol Myers Squibb, Merck Serono, MSD, Astellas, Johnson&Johnson, Astra Zeneca, Bayer, Novartis, Sanofi, Recordati, Lilly, Exelixis, Amgen, EUSApham, Roche, and Ipsen; travel expenses from Johnson&Johnson, Merck Serono, Astra Zeneca, Roche, Bayer, Bristol Myers Squibb, Amgen.

Dr A. Rodriguez-Vida reports serving in an advisory role for Pfizer, Bristol Myers Squibb, Merck Serono, MSD, Astellas, Johnson&Johnson, Astra Zeneca, Bayer, Novartis and Ipsen; receiving honoraria or travel expenses from Pfizer, MSD, Astellas, Merck Serono, Bristol Myers Squibb, Johnson&Johnson, Astra Zeneca, Bayer, Novartis and Ipsen.

Maria Natalia Gandur Quiroga has received payments or honoraria from presentations, speaker bureaus, consulting, or travel expenses from Bayer, Astellas, Novartis, MSD, Merck Serono, Adium, Pfizer, Gador, and Elea.

Thomas Büttner received honoraria from Astellas, unrelated to the present work.

Francesco Massari has received research support and/or honoraria from Advanced Accelerator Applications, Astellas, Astra Zeneca, Bayer, BMS, Janssen, Ipsen, MSD, Pfizer outside the submitted work.

Matteo Santoni has received research support and honoraria from Janssen, Bristol Myers Squibb, Ipsen, MSD, Astellas, A.A.A. and Bayer, all unrelated to the present paper.

  ***Funding/Support and role of the sponsor:*** None.
