# Supplementary material for: Role of Local Treatment to the Prostate in Patients With de Novo Low-volume Metastatic Hormone-sensitive Prostate Cancer Receiving Androgen Receptor Pathway Inhibitors
Source: Eur Urol Open Sci. 2026 Mar 30;87:16–24. doi: 10.1016/j.euros.2026.03.010 (PMC13068602; doi:10.1016/j.euros.2026.03.010)
Supplement: Supplementary Data 1 [file mmc1.docx]

**Supplementary Materials**

**Figure S1.** List of participating Countries.


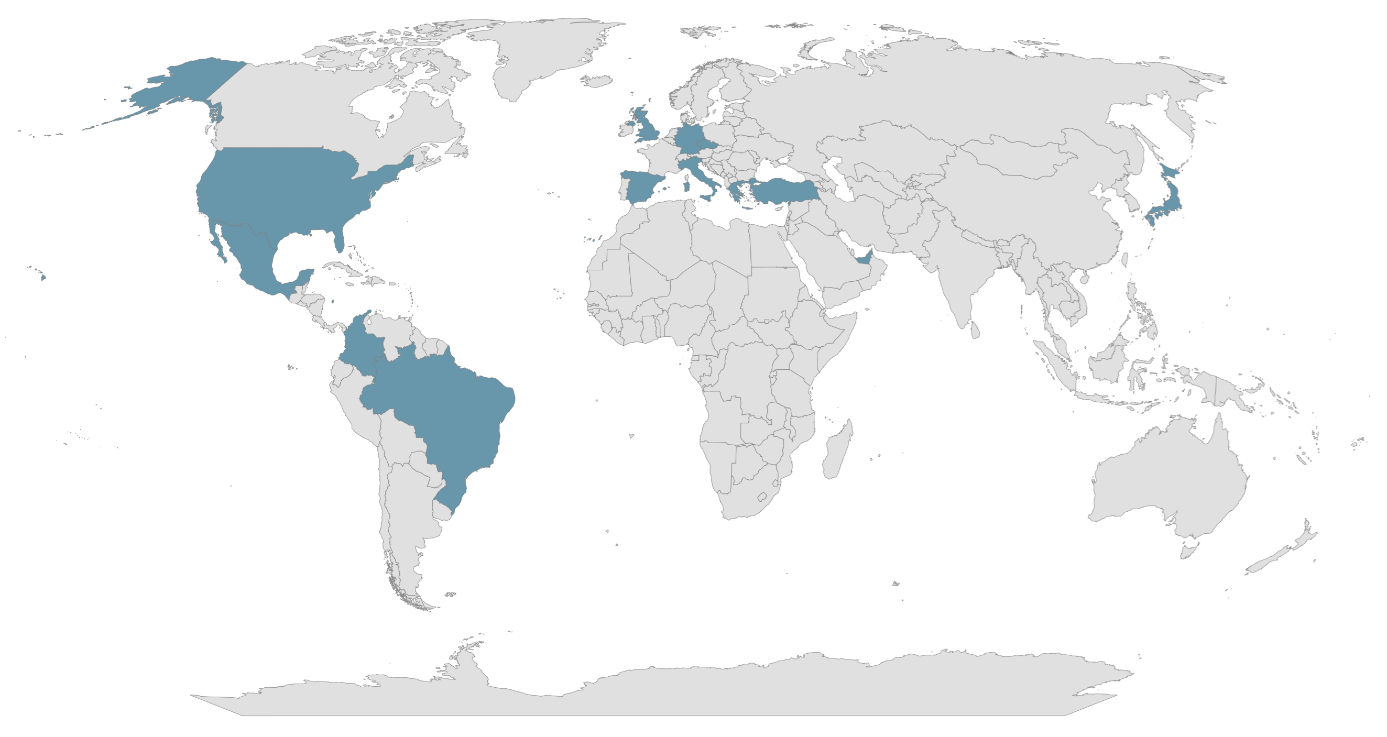


**Figure S2.** Selection process from the ARON-3 dataset.


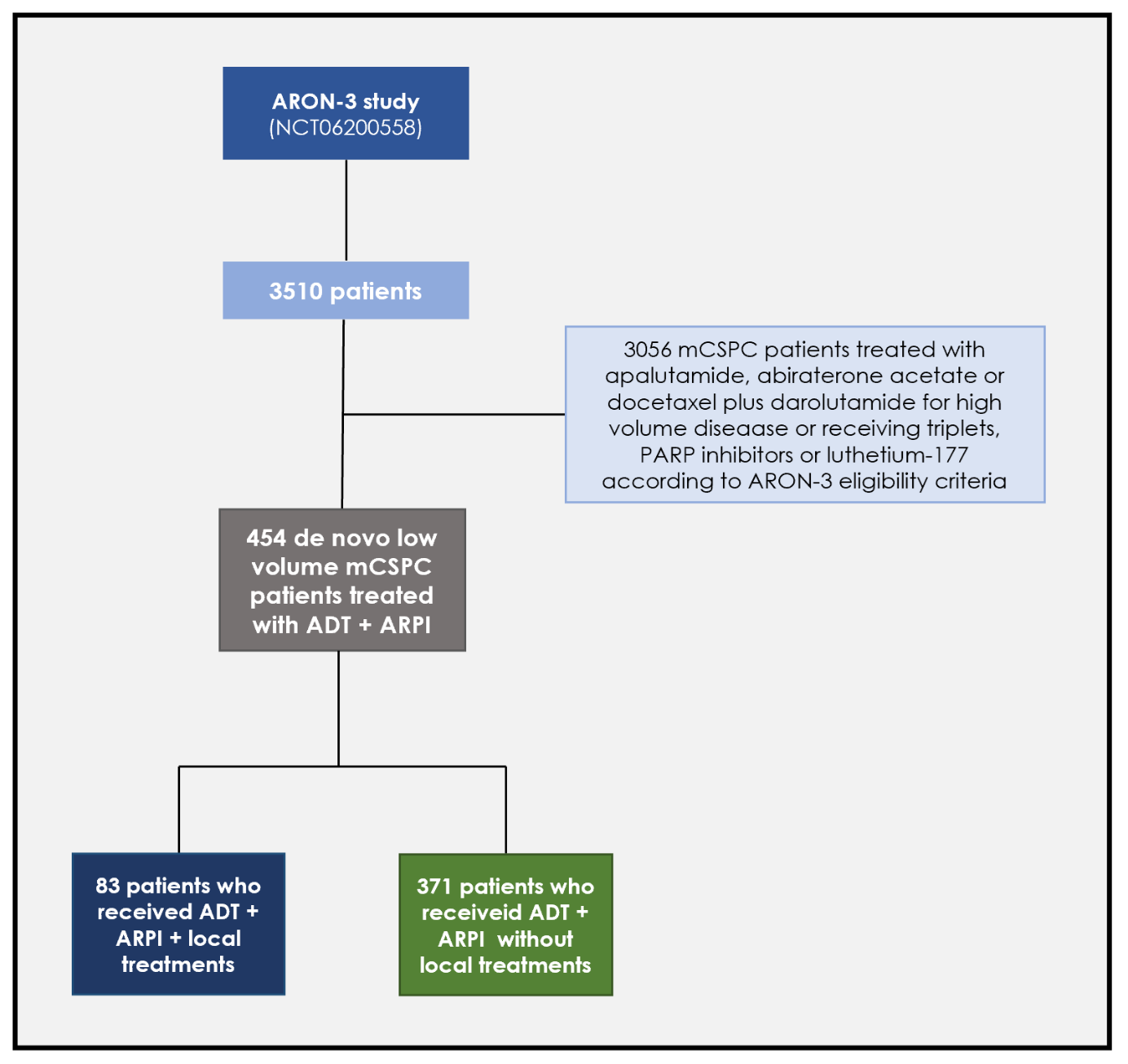


**Figure S3.** Calibration plot of the Ridge-penalized Cox Model at 24 months for Time on Treatment

**
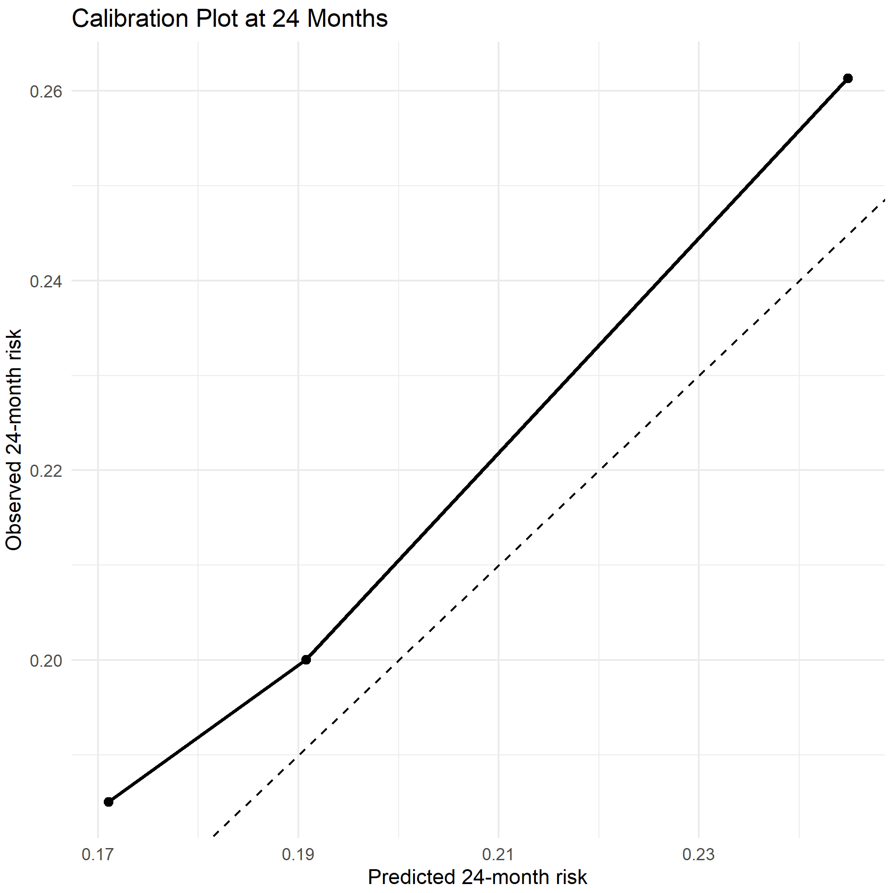
**

**Figure S4.** Calibration plot of the Ridge-penalized Cox Model at 24 months for Overall Survival

**
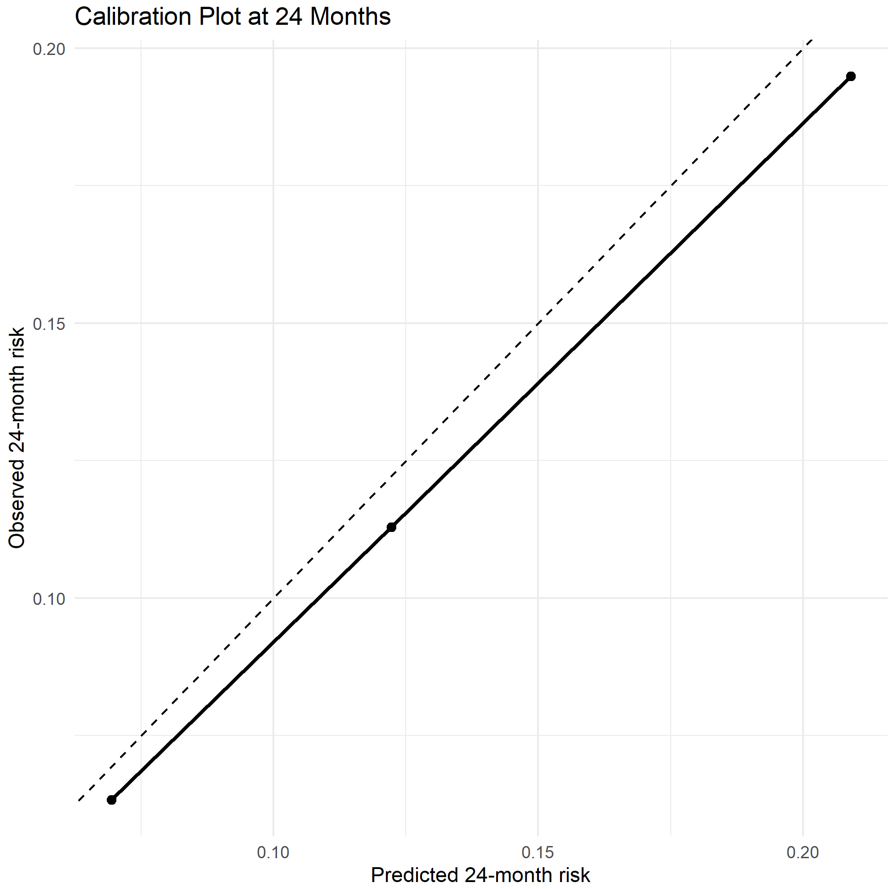
**
